# Learning from the past—A substantial number of unicompartmental knee arthroplasty revisions are potentially avoidable: A radiographic analysis of 98 cases

**DOI:** 10.1002/jeo2.70205

**Published:** 2025-04-01

**Authors:** Alexander Hoorntje, Sten van der Wilk, Iris Koenraadt‐van Oost, Rutger C. I. van Geenen

**Affiliations:** ^1^ Department of Orthopaedic Surgery & Sports Medicine, Amsterdam Movement Sciences, Amsterdam UMC University of Amsterdam Amsterdam the Netherlands; ^2^ Amsterdam Movement Sciences, Program Musculoskeletal Health Amsterdam the Netherlands; ^3^ Department of Orthopaedic Surgery Amphia Hospital Breda the Netherlands; ^4^ Foundation for Orthopedic Research, Care & Education Amphia Hospital Breda the Netherlands

**Keywords:** radiograph, revision, total knee arthroplasty, unicompartmental knee arthroplasty

## Abstract

**Purpose:**

Approximately 50% of knee osteoarthritis patients are eligible for unicompartmental knee arthroplasty (UKA), but only 10%–15% of knee replacements are UKAs. Higher UKA revision rates may prevent broader implementation. The hypothesis was that up to 40% of UKA revisions are potentially avoidable based on a radiographic analysis of indications, surgical technique and reasons for revision.

**Methods:**

Consecutive UKA revisions between 2007 and 2022 from one high‐volume UKA centre were analysed. Two independent reviewers systematically evaluated all preoperative, direct post‐operative and prerevision radiographs (anteroposterior and lateral, stress radiographs if available). Using the Oxford group criteria, adequate use of UKA indications was assessed, as well as surgical technique errors and the presence of radiographic reasons for revision. Infections were excluded.

**Results:**

Ninety‐eight revisions were included with a median time to revision of 2.2 years (interquartile range: 0.9–5.5). UKA indications were satisfied in 45%. Presence of medial bone‐on‐bone osteoarthritis was doubtful or not present in 37%. Other indications were possibly not satisfied in 18%. Post‐operative, major technical errors were identified in 7% of cases. No radiographic reason for revision was identified in 34%. Common reasons for revision were progression of lateral/patellofemoral osteoarthritis in 41%, bearing dislocation in 13% and periprosthetic fractures in 9% of cases. Uncemented fixation was associated with revision ≤2 years (*p* < 0.01), due to more periprosthetic fractures. In 47% of UKAs without preoperative bone‐on‐bone osteoarthritis, no radiographic reason for revision was identified, compared to 26% of UKAs with preoperative bone‐on‐bone osteoarthritis (*p* = 0.03).

**Conclusions:**

In conclusion, a substantial number (>40%) of UKA revisions are potentially avoidable based on the present radiographic analysis.

**Level of Evidence:**

Level III.

**Abbreviations**
ACL: anterior cruciate ligament
ASA: American Society of Anesthesiologists
BMI: body mass index
IKDC: International Knee Documentation Committee
IQR: interquartile range
MCL: medial collateral ligament
NJR: National Joint Registry
OCD: osteochondral defect
OKS: Oxford Knee Score
TKA: total knee arthroplasty
UKA: unicompartmental knee arthroplasty

## INTRODUCTION

Globally, unicompartmental knee arthroplasty (UKA) is underutilized. While estimations suggest that up to 50% of knee osteoarthritis patients who require knee replacement are eligible for UKA, studies report an overall usage of around 10%–20% [9, 28, 29, 36, 37].

This discrepancy persists despite the well‐established advantages of the UKA. Compared to total knee arthroplasty (TKA), UKA patients have shorter hospital stays and fewer complications (i.e., thromboembolic/cardiac events and early mortality) [5, 14, 20, 24, 37]. Also, UKA patients return to sport and work faster than TKA patients [17, 37, 38]. Several studies, including the randomised controlled TOPKAT trial, found UKA to have lower costs and higher cost‐effectiveness [1, 24]. So, while accumulating evidence shows the advantages of UKA, many in the orthopaedic community still prefer TKA. The main reason is the higher revision risk for UKA [4, 19, 25]. A meta‐analysis found that the annual revision rate for TKA was 0.49% (confidence interval [CI]: 0.41–0.58) compared to 1.07% (CI: 0.87–1.31) for medial UKA [4]. Much debate exists regarding the reasons for this increased revision risk.

Previous studies have suggested that a lower threshold for revision from UKA to TKA, compared to a revision from TKA to revision TKA, might be one reason for the increased UKA revision risk [33]. The most common reasons for UKA revision are aseptic loosening and progression of osteoarthritis [32]. Early revisions (within 6 months) are mostly due to infection, bearing dislocation and periprosthetic fractures [32]. Interestingly, Kennedy et al. from the Oxford UKA designer group, found that only 20% of UKA revisions were implanted for the recommended indications, with no signs of surgical errors and with a mechanical problem necessitating revision [15].

Clearly, these results merit further research. Therefore, a non‐designer study analysing consecutive Oxford UKA revisions was performed, including patient characteristics, and with available radiographs at all time points for each patient. The hypothesis was that up to 40% of UKA revisions could be potentially avoidable based on a radiographic evaluation.

## METHODS

### Study design

**Figure 1:**
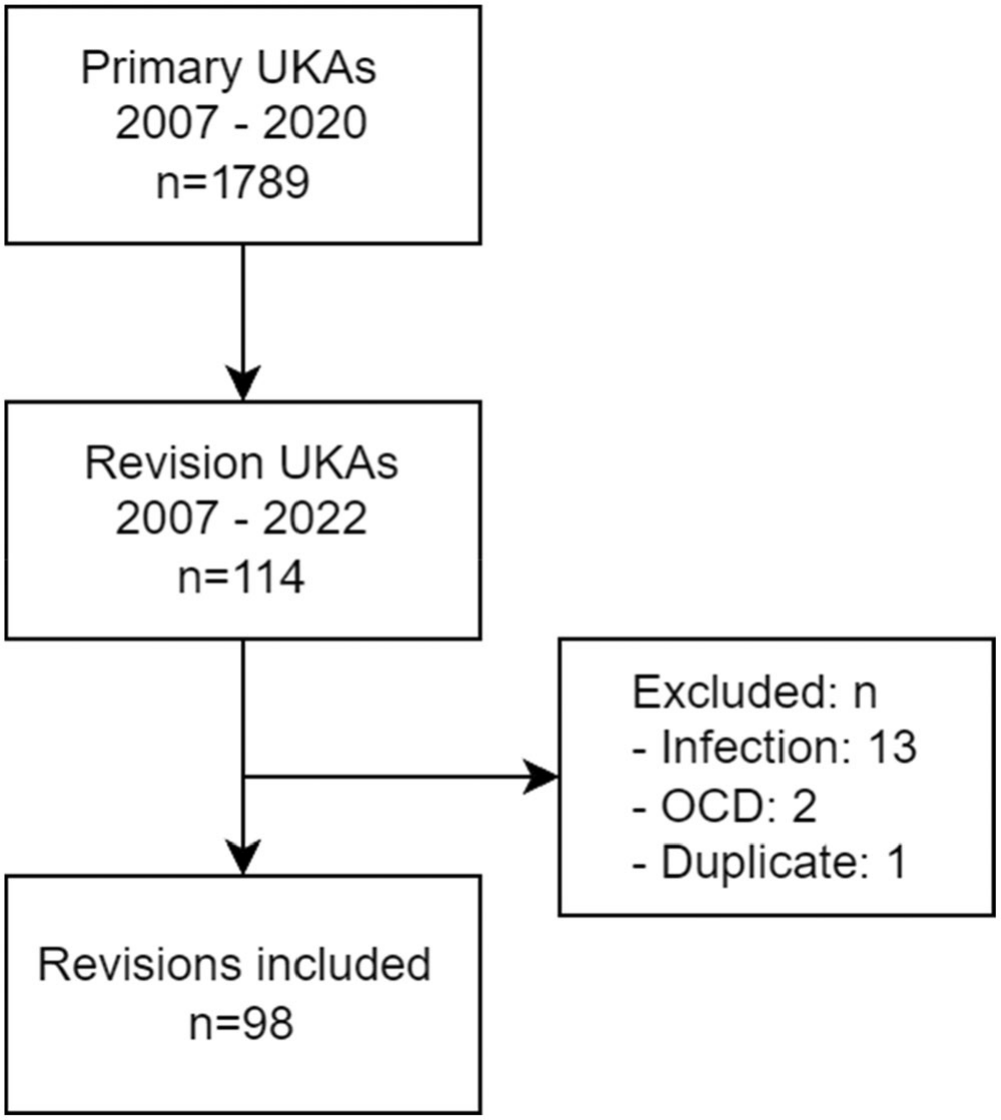
Inclusion flowchart. OCD, osteochondral defect; UKA, unicompartmental knee arthroplasty.

This single‐centre, non‐designer study was a retrospective radiographic analysis of consecutive UKAs performed between 2007 and 2020 in one large teaching hospital and a high‐volume UKA centre. In this period, 1789 primary UKAs were performed. Based on revisions registered in the Dutch Arthroplasty register between 2007 and June 2022 for this single centre, 114 UKA revisions were identified (Figure 1). Between 2007 and 2012, the cemented Oxford mobile bearing UKA was used. From May 2012, the cemented Oxford UKA was implanted with the Oxford Microplasty instrumentation [13]. From 2014, the uncemented Oxford UKA was used. The median follow‐up for the cemented UKAs was 14.4 years (interquartile range [IQR]: 10.6–15.4) and for the uncemented UKAs 6.4 years (IQR: 5.5–7.5). One case of hybrid fixation was performed, with a cemented femoral component and uncemented tibial component, due to initial malpositioning of the femoral component. After excluding revisions for infection (*n* = 13), two cases of osteochondral defects (OCDs) in relatively young patients, and one duplicate, 98 UKAs were included in the radiological evaluation. Standard standing anteroposterior and lateral radiographs are obtained preoperatively, direct post‐operatively, 6 weeks post‐operatively and 1 year post‐operatively. Stress radiographs were routinely performed during the study period. Radiographs prior to the revision were available for all patients. To evaluate the type of revision, the first post‐revision radiograph was reviewed. No re‐revisions were recorded for the study group until January 2024. Approval was obtained from the local medical ethical committee.

### Data collection

Baseline characteristics (age, sex, body mass index [BMI], smoking [yes/no], American Society of Anesthesiologists [ASA] classification and type of fixation [cemented/uncemented]) were collected from the electronic medical records. Two independent reviewers reviewed the anonymized radiographs for all patients. The Oxford UKA decision aid was used to evaluate preoperative suitability for UKA: medial bone‐on‐bone osteoarthritis; no sign of anterior cruciate ligament (ACL) insufficiency, that is, posterior erosion of the medial tibial plateau on the lateral radiograph; no sign of lateral joint space narrowing indicative of lateral osteoarthritis; no clear sign of severe patellofemoral joint space narrowing on the standard lateral radiograph [10, 11].

For the post‐operative evaluation, the criteria defined by Hurst et al., which are also explained in the Oxford UKA surgical technique manual, were used [13]. These include anteroposterior (AP) and lateral positioning of the femoral and tibial components, depth of tibial saw cuts, and evidence of bearing impingement. In line with a previous study by Kennedy et al., observed technical errors were graded as major if they were likely to cause implant failure (e.g., severe under‐sizing of tibial implant), or as minor if they were less likely to cause implant failure [15].

After scoring all radiographs, a consensus meeting was held to evaluate differences and reach consensus. In case of discussion, the senior author had the final vote. A random sample of 10% of cases was separately evaluated by the senior author, with an almost perfect agreement.

### Data analyses

Because of the descriptive nature of the study, no sample size calculation was performed. Cohen's Kappa was calculated for the agreement between the reviewers (moderate 0.41–0.60; substantial/good 0.61–0.80; almost perfect 0.81–1.00). Baseline characteristics were analysed using descriptive statistics. Possible errors in the preoperative selection and post‐operative surgical errors, as well as cases where no radiological explanation for the revision was observed, are reported as numbers with percentages. The association between the presence of preoperative bone‐on‐bone osteoarthritis (yes/no) and a pre‐revision radiographic reason for revision (present/not present) was analysed using the chi‐square test [8]. To analyse possible factors associated with early revision (≤2 years [32]) for all causes, univariate analysis was performed. Also, a logistic regression analysis was conducted to examine whether the presence of a surgical technique error (no error, minor error or major error) was a significant predictor of revision within 2 years. Because of the difference in follow‐up between the cemented and uncemented groups, the *Z*‐score test for proportions was used to analyse the risk of early revision between the cemented and uncemented groups. All analyses were performed using SPSS (Version 28.0).

The STROBE guidelines for observational studies were followed for the present study [7].

## RESULTS

A total of 98 UKA revisions were included in the analysis (Figure 1). Cohen's kappa for the agreement between both reviewers was 0.77 for the preoperative radiographs, 0.46 for the post‐operative radiographs, and 0.98 for the pre‐revision radiographs. The median time to revision was 2.2 years (Table 1).

**Table 1:** Baseline characteristics.

	UKA revisions (*n* = 98)
Sex, female (%)	63 (64%)
Age at index surgery, years (mean ± SD)	64.9 ± 9.4
Side, right (%)	52 (53%)
BMI, kg/m^2^ (mean ± SD)	30.1 ± 4.3
ASA classification, *n* (%)	
I	11 (11%)
II	62 (63%)
III	25 (26%)
Smoking, yes (%)	12 (12%)
Fixation, *n* (%)	
Cemented	57 (58%)
Hybrid	1 (1%)
Uncemented	40 (41%)
Age at revision surgery, years (mean ± SD)	68.3 ± 9.4
Time to revision, years (median + IQR)	2.2 (0.9–5.5)

Abbreviations: ASA, American Society of Anesthesiologists; BMI, body mass index; IQR, interquartile range; SD, standard deviation; UKA, unicompartmental knee arthroplasty.

**Figure 2:**
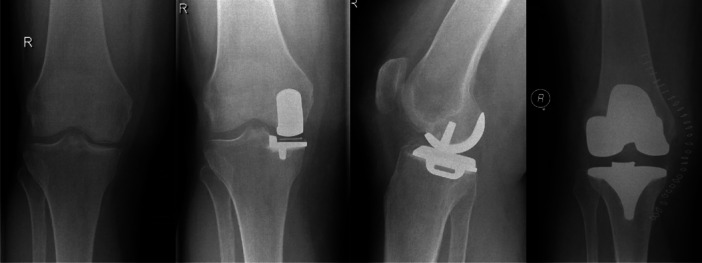
Example of a patient without bone‐on‐bone osteoarthritis on the preoperative radiograph. The post‐operative radiographs show a well‐aligned UKA. This patient was revised for unexplained pain. UKA, unicompartmental knee arthroplasty.

In 44 patients (45%), the indications for medial UKA were definitely satisfied (Table 2). The presence of medial bone‐on‐bone osteoarthritis was doubtful or definitely not present in 36 patients (37%; Figure 2). Patellofemoral joint space narrowing (13%) and lateral joint space narrowing (3%) were also observed. In two patients (2%), ACL insufficiency was suspected because of a posterior wear pattern on the lateral radiographs.

**Table 2:** UKA suitability according to preoperative radiographic evaluation.

	Preoperative radiographs (*n* = 98)
Indications satisfied	44 (45%)
Presence of medial bone‐on‐bone OA doubtful or definitely not present	36 (37%)
Patellofemoral joint space narrowing	13 (13%)
Lateral joint space narrowing	3 (3%)
Possible ACL insufficiency	2 (2%)

Abbreviations: ACL, anterior cruciate ligament; OA, osteoarthritis; UKA, unicompartmental knee arthroplasty.

**Figure 3:**
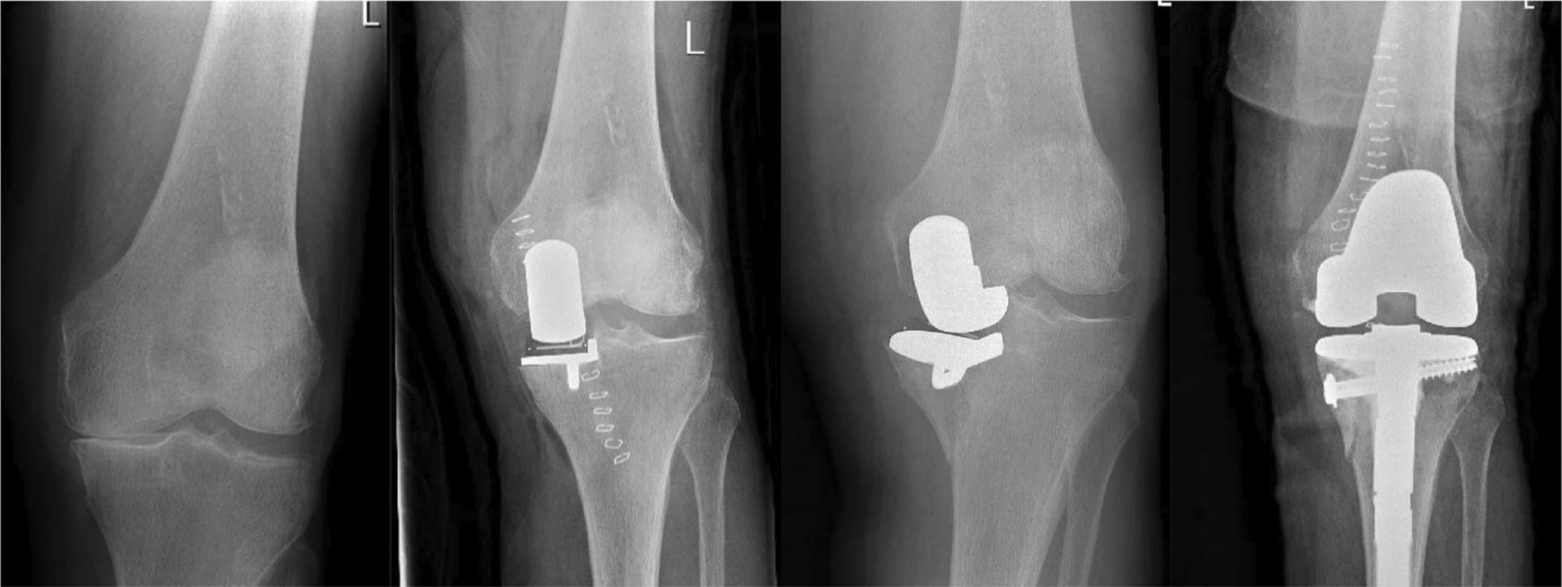
Preoperative radiograph shows bone‐on‐bone medial osteoarthritis. The post‐operative radiograph suggests a deep vertical tibial saw cut, leading to a periprosthetic fracture of the tibial component. This patient was revised with a tibial revision stem and screw augmentation.

Post‐operative radiographs showed seven major technical errors, all related to the tibial cut (Table 3; Figure 3). Sixty‐seven minor technical errors were seen, often related to the vertical tibial cut being too medial, resulting in sizing issues and component gap errors. Malrotation of tibial (eight errors) or femoral (one error) components was less frequently seen. In two cases, a corpus liberum (cement) was seen on the post‐operative radiograph.

**Table 3:** Evaluation of technical errors on post‐operative radiographs.^a^

	Post‐operative radiographs (*n* = 98)
No error identified	29 (30%)
*Major*	*7 (7%)*
Tibial cut errors	1 (1%)
Tibial component under sizing	5 (5%)
Excessive posterior tibial slope (>12°)	1 (1%)
*Minor*	*67 (68%)*
Tibial cut errors	25 (25%)
Tibial component varus/valgus malalignment (>5°)	8 (8%)
Femoral cut errors	13 (13%)
Femoral component varus/valgus malalignment (>0°)	1 (1%)
Component malsizing	8 (8%)
Component gap error	10 (10%)
Corpus liberum	2 (2%)

Abbreviation: UKA, unicompartmental knee arthroplasty.

^a^ More than one error per UKA could be scored.

**Figure 4:**
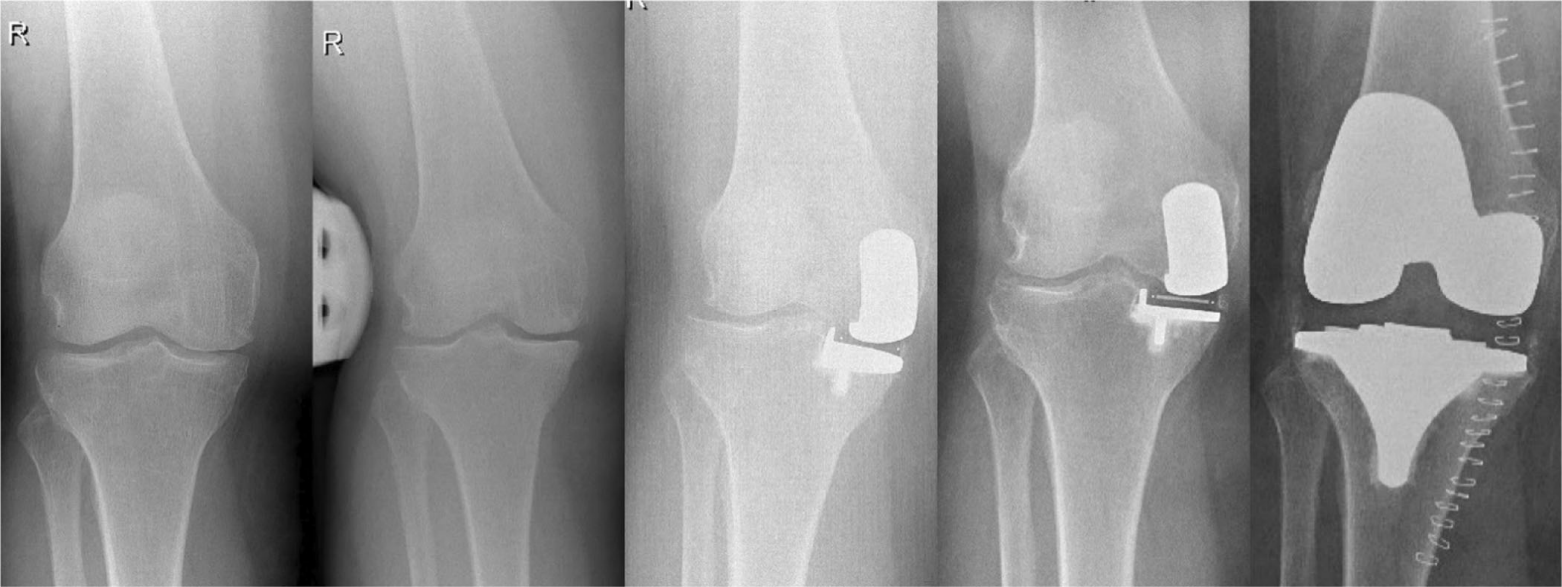
Preoperative radiographs show near bone‐on‐bone medial osteoarthritis (OA), and possibly slight joint space narrowing of the lateral compartment on the stress radiograph. The post‐operative radiograph shows no abnormalities. The pre‐revision radiograph shows lateral OA progression.

On 33 pre‐revision radiographs (34%), no reason for revision was seen (Table 4). The most common cause for revision was progression of lateral and/or patellofemoral osteoarthritis (41%; Figure 4). Bearing dislocation was seen in 13 patients (13%), aseptic loosening in 10 patients (10%) and periprosthetic fractures in 9 patients (9%). Periprosthetic tibial fractures occurred in two cemented UKAs (4%) and seven uncemented UKAs (18%, *p* = 0.03). Less common causes included polyethylene wear, ACL or medial collateral ligament insufficiency, and a corpus liberum (Table 4). In 17 out of 36 cases (47%) without preoperative bone‐on‐bone osteoarthritis, no radiographic reason for revision was identified, compared to 16 out of 62 cases (26%) with preoperative bone‐on‐bone osteoarthritis (*p* = 0.03). Not meeting the preoperative UKA criteria was not associated with early revision <2 years (*p* = 0.44; Table 5). Fixation method was the only factor associated with an early revision. More early revisions were seen in the uncemented group than in the cemented group (*p* < 0.01; Table 5).

**Table 4:** Evaluation of reasons for revision on pre‐revision radiographs.

	Pre‐revision radiographs (*n* = 98)
No reason for revision identified	33 (34%)
Lateral and/or patellofemoral OA progression^a^	41 (41%)
Bearing dislocation	13 (13%)
Aseptic loosening	10 (10%)
Periprosthetic fracture	9 (9%)
MCL insufficiency	3 (3%)
Polyethylene wear	2 (2%)
ACL insufficiency	1 (1%)
Corpus liberum/impingement	1 (1%)

Abbreviations: ACL, anterior cruciate ligament; MCL, medial collateral ligament; OA, osteoarthritis.

^a^ Both lateral and patellofemoral OA progression could be present in the same patient.

**Table 5:** Univariate analysis of factors associated with revision ≤2 years of primary UKA.

	Revision ≤2 years (*n* = 47)	Revision >2 years (*n* = 51)	*p* value
Sex, female (%)	28 (60%)	35 (69%)	0.35^a^
Age at index surgery, years (mean ± SD)	66.3 (±9.9)	63.6 (±8.8)	0.08^b^
BMI, kg/m^2^ (mean ± SD)	29.6 (±4.0)	30.5 (±4.5)	0.17^b^
ASA classification, *n* (%)			0.61^a^
I	5 (11%)	6 (12%)	
II	32 (68%)	30 (59%)	
III	10 (21%)	15 (29%)	
Smoking, yes (%)	4 (9%)	8 (16%)	0.20^a^
Fixation, *n* (%)			**<0.01** c
Cemented	20 (42%)	38 (74%)	
Uncemented	27 (58%)	13 (26%)	
Preoperative UKA criteria satisfied, *n* (%)			0.44^a^
Yes	23 (49%)	21 (41%)	
No	24 (51%)	30 (59%)	

Abbreviations: ASA, American Society of Anesthesiologists; BMI, body mass index; SD, standard deviation; UKA, unicompartmental knee arthroplasty.

a Chi‐square test.

b Independent samples *t* test.

^c^ *Z*‐score test for the difference in proportion for revision ≤2 years between the cemented and uncemented groups.

Patients without a radiographic reason for revision were younger, but the other baseline variables did not differ between the UKAs with and without a radiographic reason for revision (Table 6).

**Table 6:** Univariate analysis of factors associated with UKA revision, with and without a radiographic reason for revision.

	Radiographic cause for revision, yes (*n* = 65)	Radiographic cause for revision, no (*n* = 33)	*p* value
Sex, female (%)	45 (69%)	18 (55%)	0.15^a^
Age at index surgery, years (mean ± SD)	66.5 (±10.0)	61.7 (±7.1)	**<0.01** b
BMI, kg/m^2^ (mean ± SD)	30.1 (±4.3)	30.0 (±4.4)	0.95^b^
ASA classification, *n* (%)			0.21^a^
I	6 (9%)	5 (15%)	
II	39 (60%)	23 (70%)	
III	20 (31%)	5 (15%)	
Smoking, yes (%)	7 (11%)	5 (15%)	0.46^a^
Fixation, *n* (%)			0.28^c^
Cemented	36 (55%)	22 (67%)	
Uncemented	29 (45%)	11 (33%)	
Preoperative UKA criteria satisfied, *n* (%)			0.73^a^
Yes	30 (46%)	14 (42%)	
No	35 (54%)	19 (58%)	

Abbreviations: ASA, American Society of Anesthesiologists; BMI, body mass index; SD, standard deviation; UKA, unicompartmental knee arthroplasty.

^a^ Chi‐square test.

^b^ Independent samples *t* test.

^c^ *Z*‐score test.

The logistic regression model analysing the effect of surgical errors on early revisions did not significantly improve over the null model (χ² = 0.273, *p* = 0.87), indicating that the presence of a surgical technique error was not a significant predictor of early revision (Table 7). The overall model explained very little variance in revision outcomes (Nagelkerke *R* ^2^ = 0.004).

**Table 7:** Logistic regression analysis for the effect of minor and/or major surgical technique errors on the risk of revision ≤2 years of primary UKA.

Predictor	*B*	SE	Exp(*B*) (odds ratio)	95% CI for Exp(*B*)	*p* value
Minor error vs. No error	−0.198	0.45	0.82	0.34–1.98	0.66
Major error vs. No error	−0.357	0.85	0.70	0.13–3.88	0.68

Abbreviations: CI, confidence interval; SE, standard error; UKA, unicompartmental knee arthroplasty.

## DISCUSSION

The findings of the present radiographic analysis of UKA revisions support the hypothesis that up to 40% of UKA revisions could be potentially avoidable. First, in only 45% of cases, the preoperative criteria for Oxford UKAs were definitely satisfied. The most important preoperative criterion, the presence of medial bone‐on‐bone osteoarthritis, was not found in 37% of cases. These patients were almost twice as likely to be revised without radiographic evidence of implant failure in the present cohort. Major post‐operative errors were relatively rare (7%), and did not seem to influence the risk of early revision, but pre‐revision radiographs showed no clear reason for revision in 34% of cases. Finally, revision for periprosthetic fractures was more likely in the uncemented group.

Kennedy et al. performed a radiographic analysis of 107 revised Oxford UKAs in the National Joint Registry (NJR) [15]. The authors found that inappropriate patient selection was present in 30% of cases, major surgical errors were seen in 6% of cases, and no radiographic evidence of joint failure prior to the revision was found in 67% of cases. A limitation of their approach was that demographic data were not available. Also, matching radiographs for individual patients were only present in 71%–91% of cases. Still, the authors concluded that the high UKA revision rate in the UK registry is likely, or at least partly, due to inappropriate patient selection and inappropriate reasons for revision.

To emphasise the necessity of meeting preoperative UKA criteria, Oxford UKA in patients without bone‐on‐bone osteoarthritis on x‐rays will likely result in unsatisfactory functional results and worse survival rates [8, 22, 27, 35, 39]. In a matched cohort of Oxford UKAs performed in patients with bone‐on‐bone osteoarthritis and with partial cartilage loss, patients in the partial cartilage loss group had lower Oxford Knee Scores (OKS) and American Knee Society Scores at 1, 2 and 5 years [8]. Patients in the partial cartilage loss group were three times more likely to undergo further surgery than those in the bone‐on‐bone osteoarthritis group. A later study by Carlson et al. on 271 UKAs with severe medial osteoarthritis (International Knee Documentation Committee [IKDC] Grade D) suggested that a cut‐off of ≤2 mm of remaining cartilage in the medial compartment did not compromise revision‐free survival at 8 years [3]. Yet, this study did not report the routine use of varus‐valgus stress radiographs. It is presumable that bone‐on‐bone osteoarthritis would be present on stress radiographs in almost all of the cases with IKDC Grade D osteoarthritis. In this study, revision without the presence of a radiographic reason, that is, for unexplained pain or an unsatisfactory functional result, was almost two times as likely in patients without than in patients with preoperative bone‐on‐bone osteoarthritis (47% vs. 26%). This finding stresses the importance of strict adherence to the well‐established UKA indications [9, 10].

Next, major surgical errors, as seen on post‐operative radiographs, were relatively uncommon in the study group (7%). Major errors were almost exclusively associated with the tibial cut and tibial component placement. However, the nine cases of periprosthetic fractures may also have been associated with the surgical technique, even if the direct post‐operative radiograph did not reveal saw‐cut errors [2, 14]. A periprosthetic fracture is a rare complication after UKA, with an estimated incidence rate of up to 1% [14]. Associated patient factors include high BMI, older age, female sex and decreased bone mineral density [2, 14]. Associated surgical factors include a deep vertical cut into the posterior proximal tibia, insufficient or inappropriate keel slot preparation, use of a heavy hammer, multiple pin holes (>2) for the tibial cutting jig, a small tibial component size, and valgus inclination of the tibial component [2, 14]. A trick to prevent a deep vertical tibial cut is to start with the horizontal cut and place a stop (angel wing, saw blade and saw stop) in this horizontal saw cut before making the vertical saw cut. Also, changing the diameter of the reciprocating keel saw to a thicker saw blade may lower the occurrence of periprosthetic fractures, without increasing the risk of tibial component loosening [16]. One could consider performing a cemented tibial component in small, female patients and patients with osteoporosis. However, prospective studies are needed to establish the effect of such an algorithm on the occurrence of periprosthetic fractures after UKA. Finally, the emergence of kinematic alignment techniques for UKA is noteworthy since the authors state that kinematic alignment could prevent surgical errors related to implant alignment [23, 30]. To date, no RCTs have been published comparing mechanical and kinematic alignment, which makes the comparison between both techniques for UKA difficult [30], and future studies are needed.

Finally, UKA revision cases without clear radiographic evidence of implant failure should be critically reviewed. Although patient‐reported data after UKA revision for unexplained pain are sparse [15], TKA literature convincingly showed that patients with unexplained pain and without any recognised pathology should be treated conservatively since they may improve over a period of time and rarely do so after a revision operation [34]. Several reasons may have contributed to the high percentage (34%) of these cases in the present study. First, during the early years of the study period, the use of bone scintigraphy to diagnose aseptic loosening was relatively common. Scans were also performed in cases of unexplained pain within 2 years of index surgery. However, studies have shown the limited value of bone scintigraphy for diagnosing early loosening after TKA and UKA, since physiological uptake can be seen up to 2 years after the index surgery [6, 12, 31]. Therefore, a number of patients in the study cohort may have been wrongfully revised for assumed aseptic loosening, although the analysis of radiographs did not allow for reporting an exact number of patients.

Additionally, thresholds for revision following UKA are lower than for TKA, because the revision operation for a UKA is considered easier and less invasive than a TKA revision. Tay et al. performed a large propensity‐matched study with data from the New Zealand Joint Registry. Compared with TKA, the relative risk for UKA revision for ‘unknown’ reasons was 2.5 times higher between 6 months and 10 years of follow‐up [33]. Also, more UKA patients with poor functional results, as reported by the OKS, underwent revision between 6 months and 5 years of follow‐up. UKA revisions were associated with a higher incidence of different failure modes than TKA, namely bearing dislocations and disease progression. These findings suggest that a lower clinical threshold for UKA revision may partly explain the higher revision risk found in many registries.

Finally, regarding general recommendations to lower UKA revision rates, the importance of surgeon caseload and UKA proportion as part of the surgeon's knee arthroplasty practice has been well established [11, 26, 28]. Analysis of the NJR revealed a significant improvement in 10‐year survival rates for surgeons with an annual caseload of >10 UKAs [26]. The 10‐year survival with revision as end point was 84% for low caseload surgeons and 90% for high caseload surgeons. Regarding UKA proportion, Liddle et al found that optimal UKA results are obtained with UKA usage between 40% and 60%. Surgeons who meet this proportion had 5‐year survival rates of 96% in the NJR. In contrast, surgeons with UKA usage of 5% had a 90% 5‐year survival rate [21]. Unfortunately, the analysis by Klasan et al. of UK, Australian and New Zealand registry data showed that more than 50% of knee surgeons in each registry performed UKA in less than 5% of cases [18].

A limitation of this study is the heterogeneity in available perioperative radiographs and their quality, which sometimes hampers adequate assessment. Also, the relatively high intra‐rater variability for the direct post‐operative radiographs was notable. This may be explained by the extensive checklist as provided by the Oxford UKA manufacturer, including 17 items for the direct post‐operative radiographs. Sometimes, reasons for revision might not be visible on static radiographs (instability and bearing subluxation), although this likely accounts for a minority of cases. Finally, a radiographic analysis does not allow for comparisons of patient‐reported and functional outcomes. Thus, it cannot be concluded that revisions without radiographic evidence of implant failure were unnecessary, because patients may have experienced significant improvements after their revision. Yet, previous data on TKAs have shown that revisions for causes such as unexplained pain rarely result in satisfied patients. Still, a future study comparing patient‐reported outcomes between UKA revisions with and without radiographic evidence of implant failure would be interesting.

## CONCLUSION

In conclusion, a substantial number (>40%) of UKA revisions are potentially avoidable based on the present radiographic analysis.

## AUTHOR CONTRIBUTIONS

**Alexander Hoorntje**: Conceptualisation; methodology; formal analysis; writing—original draft; funding acquisition. **Sten van der Wilk**: Formal analysis; investigation; visualisation; writing—review and editing. **Iris Koenraadt‐van Oost**: Resources; writing—review and editing. **Rutger van Geenen**: Conceptualisation; methodology; writing—review and editing; supervision. The first draft of the manuscript was written by Alexander Hoorntje, and all authors commented on previous versions of the manuscript. All authors read and approved the final manuscript.

## CONFLICT OF INTEREST STATEMENT

Rutger van Geenen is a paid consultant for Zimmer. The remaining authors declare no conflicts of interest.

## ETHICS STATEMENT

Ethical approval was waived by the local ethics committee in view of the retrospective nature of the study and because all the procedures being performed were part of the routine care.

## Data Availability

The data sets used and/or analysed during the current study are available from the corresponding author on reasonable request.
